# Exploring the Impact of α-Amylase Enzyme Activity and pH on Flavor Perception of Alcoholic Drinks

**DOI:** 10.3390/foods12051018

**Published:** 2023-02-27

**Authors:** Maria João Santos, Elisete Correia, Alice Vilela

**Affiliations:** 1 Department of Agronomy, University of Trás-os-Montes e Alto Douro, 5001-801 Vila Real, Portugal; 2 Center for Computational and Stochastic Mathematics (CEMAT), Department of Mathematics, University of Trás-os-Montes and Alto Douro, 5001-801 Vila Real, Portugal; 3 Chemistry Research Centre (CQ-VR), Department of Agronomy (DAgro), School of Agrarian and Veterinary Sciences (ECAV), University of Trás-os-Montes e Alto Douro, 5001-801 Vila Real, Portugal

**Keywords:** panel of tasters, pH, α-amylase, lipase, salivary parameters

## Abstract

The introduction of a drink in the mouth and the action of saliva and enzymes cause the perception of basic tastes and some aromas perceived in a retro-nasal way. Thus, this study aimed to evaluate the influence of the type of alcoholic beverage (beer, wine, and brandy) on lingual lipase and α-amylase activity and in-mouth pH. It was possible to see that the pH values (drink and saliva) differed significantly from the pH values of the initial drinks. Moreover, the α-amylase activity was significantly higher when the panel members tasted a colorless brandy, namely Grappa. Red wine and wood-aged brandy also induced greater α-amylase activity than white wine and blonde beer. Additionally, tawny port wine induced greater α-amylase activity than red wine. The flavor characteristics of red wines due to skin maceration and the contact of the brandy with the wood can cause a synergistic effect between beverages considered “tastier” and the activity of human α-amylase. We can conclude that saliva-beverage chemical interactions may depend on the saliva composition but also on the chemical composition of the beverage, namely its constitution in acids, alcohol concentration, and tannin content. This work is an important contribution to the e-flavor project, the development of a sensor system capable of mimicking the human perception of flavor. Furthermore, a better understanding of saliva–drink interactions allow us to comprehend which and how salivary parameters can contribute to taste and flavor perception.

## 1. Introduction

From an early age, taste and aroma have been known for walking together in a single direction [1]. It is the combination of that leads to the recognition of the most varied sensory experiences to which the human being’s palate is constantly subjected.

Trigeminal sensations—or “mouthfeel”—are defined as “a group of sensations that is characterized by the tactile response in the mouth” [2] and described as “tactile properties (sensation) perceived from the moment that food or drink—solid, semi-solid or liquid—is placed in the mouth until swallowed” [3]. These sensations, together with the taste and olfactory sensations (ortho- and retronasal perception), contribute to the flavor of the food/drinks that are essential in the acceptance of these products by consumers [4]. Understanding the factors affecting flavor perception may provide clues to drive food consumption toward keeping a proper nutritional status [5].

Apart from food characteristics, flavor perception is strongly influenced by oral physiology, namely saliva [5]. Saliva’s constitution is approximately 99% water, and the rest is inorganic and organic compounds [6,7]. The pH of saliva is between 6.2 and 7.4 [8]. Being slightly acidic or basic, saliva is a highly viscoelastic fluid and has unique properties that facilitate chewing, digestion, homeostasis, and flavor perception, among many other things [9,10]. Moreover, in addition to dilution, the buffering capacity of saliva has also been reported to decrease the response to acid stimulation, although the concentration of organic acids in food and drinks and, therefore, titratable acidity also contributes to sourness [11]. 

The different constituents of saliva are responsible for the interaction and formation of new compounds within the oral cavity [6], and salivary proteins are of the utmost importance since they fulfill various roles, such as oral digestion (amylases and lipases), neutralization of toxic molecules (proline-rich proteins and histatins), defense against microorganisms (immunoglobulins and peroxidases), lubrication of the oral cavity (mucins), and the transport of flavor molecules (lipocalins) [12]. These enzymes are secreted by the salivary glands or originate from the lysis of desquamated epithelial cells [12]. Given the presence of microbiota in the mouth, some enzymes can be of bacterial origin [13]. The total protein concentration in saliva is around 0.2–2.0 mg/mL, and more than 4000 different proteins are present [14]. Among others, two enzymes may contribute to flavor-taste perception: lipase and α-amylase. Several researchers state that orally expressed lipases might hydrolyze triacylglycerols and, consequently, release non-esterified fatty acids (NEFA). However, fatty acids are poorly soluble in aqueous solvents, which could prevent their access to their sensory receptors. It has also been hypothesized that salivary proteins could play a role in the transport of NEFA in the mouth [5]. The α-amylase enzyme is the main protein in human saliva and is responsible for catalyzing the hydrolysis of 1,4-glycosidic bonds in starch and other polysaccharides, such as glucose and maltose, which are smaller sugars that can be detected by the sweet receptors in the mouth [15,16]. Moreover, the secretion of salivary proteins, such as α-amylase, has been reported to be dependent on the diet [17], and the salivary proteome and glucose levels have been related to sweet taste sensitivity in young adults [18] and α-amylase concentrations in healthy children [19].

Regarding aroma perception, namely the retro-nasal pathway, volatile molecules, before being detected by olfactory receptors, are influenced by factors such as body temperature and saliva pH, allowing the identification of different and equally important aromas [20,21]. When volatiles are released from food into the saliva phase, chemical and biochemical reactions occur, which could affect the volatile concentration and retro-nasal aroma perception [22]. Several authors note that salivary proteins, such as mucins and α-amylase, can ‘trap’ aroma compounds depending on their structure [23]. Some aroma compounds can be individually metabolized in the oral cavity, leading to the formation of new metabolites with different odor thresholds [24,25]. Moreover, the persistence of an aroma compound also depends on its metabolism in the oral cavity, as referred to by Muñoz-Gonzalez et al. [26], who found that the perceived aroma intensity of the compounds that are metabolized in the oral cavity decreases faster than that of the nonmetabolized compounds. Furthermore, aroma compounds may adsorb onto the mucosal pellicle, while the aggregation of the mucosal pellicle by tannins may disturb these interactions [27].

The main objective of this work was to study the differences in pH and the variations that occur in saliva enzyme activity, lipase, and α-amylase, depending on the type of alcoholic beverage that is consumed. This work is an important contribution to the e-flavor project, the development of a sensor system capable of mimicking the human perception of flavor in alcoholic drinks, namely wine. Furthermore, a better understanding of the saliva–drink interactions allow us to comprehend which and how salivary parameters can contribute to taste and flavor perception. To this end, we analyzed the enzymatic activity of lipase and α-amylase and pH changes before and after the contact of alcoholic drinks with the tasters’ saliva.

## 2. Materials and Methods

### 2.1. Ascending Method of Limits for Primary Taste Detection Thresholds in Aqueous and Hydroalcoholic Solutions

Aqueous and hydroalcoholic (12%, *v*/*v*) solutions associated with each basic taste (sweet, salty, acid, and bitter) were prepared to determine the sensory and perception threshold of the tasters. For each basic taste attribute, seven aqueous solutions with different concentrations were prepared (Table S1). For the acid taste attribute, acids were tested in separated solutions (Table S2). In each aqueous solution, 0.5 L of water (bottled water—Caldas of Penacova) was used. The procedure was similar to the preparation of the 12% hydroalcoholic solutions (Tables S3 and S4). Before preparing the solutions, a mother solution was prepared with water and ethanol, with a concentration of 12% (*v*/*v*), simulating the alcohol level of table wines.

The final pH of each of the solutions at different concentrations was also measured (Table S2).

The solutions were presented to the tasters in ascending order of concentration [28,29] at a temperature of 20 °C. After tasting, the tasters had to indicate on the tasting sheet if the solution caused any sensation—sensation threshold—and identify the taste in question—perception threshold.

### 2.2. Tasting Panel Characterization and Laboratory Conditions

For this study, two different panels were used. The first panel (P1) of tasters was composed of 11 individuals, of which 10 were female and only 1 was male. The ages of the panel were between 39 and 59 years, and they had previously participated in assessments of food and beverages in several published works [30,31,32]. The second panel (P2) of tasters was composed of 19 individuals, of which 4 were female and 15 were male. The ages of the panel were between 20 and 50 years, and they were the untrained panel. In order not to impair the results of the sensory evaluation, the tasters were non-smokers and were told not to use any type of perfume or other cosmetics with a strong aroma and not to eat or drink anything, except water, for one hour before the start of the tests.

All evaluations were conducted from 4:00 to 6:00 p.m. and took place in individual tasting booths in a sensory laboratory [33] using the 21.5 cl transparent ISO wine-tasting glasses [34]. Sessions were carried out under controlled conditions at 20 °C (±2 °C) and relative humidity of 60% (±20%).

A volume of 20 mL was used in all the tasting sessions. This volume was chosen to make it possible for the tasters to put all the liquid in their mouths for 10 s and then spit it back into the glass to be collected and stored.

### 2.3. Methodology for pH Changes and Enzyme Activity Evaluation

#### 2.3.1. Solutions and Alcoholic Beverages

Each basic taste was associated with an aqueous solution with a certain concentration of the taste in question. For bitter taste and astringency in the lipase and α-amylase tests, we choose to use a tannin solution and not quinine, as tannin is an alcoholic drink component important for bitter taste and astringency.

The selection of concentration was based on the concentration where most tasters perceived and identified the taste (Table 1). The procedure for the preparation of the hydroalcoholic solutions (ethanol 12%, *v*/*v*) was identical to what was described above.

Regarding alcoholic beverages, each taster from panel one (P1) was presented with one beer (blonde beer, with an alcohol content of 4.9% (*v*/*v*) and pH 4.34), two wines (white wine with an alcohol content of 13.0% (*v*/*v*) and pH 3.37, and red wine with an alcohol content of 13.9% (*v*/*v*) and pH 3.87) and two brandies (a colorless brandy and a wood-aged brandy with alcohol contents of 41.0% (*v*/*v*) and 40.0% (*v*/*v*), respectively, and pH 4.30 and 3.93, correspondingly). To P2 (panel two) were presented two wines, one red wine and one port wine, with alcohol contents of 13.5% and 19% (*v*/*v*), respectively. 

The wines, beer, and brandies were stored in a climate-controlled dark cellar maintained at 11 °C ± 1 °C. The day before the sensory sessions, the white wine and beer were stored at 4 °C ± 2 °C in a fridge.

#### 2.3.2. Tasting Procedure and Saliva Collection

In the first part of the work, for P1, saliva was collected from the members of the panel during two tasting sessions, one for the aqueous and hydroalcoholic solutions and another for the alcoholic drinks (beer, wine, and brandy). In the second part of the work, for P2, saliva was collected after tasting two alcoholic beverages, namely red wine, and port wine. The procedure was similar in both sessions.

The solutions described in Table 1 and the beverages in both procedures were presented to the tasting panel in codded 21.5 cl transparent ISO wine-tasting glasses [34] with a quantity sufficient for the taster to place in the mouth (20 mL). The tasting procedure was explained, and it was requested that the tasters place the sample in their mouths, swirl the liquid through the mouth cavity, and wait for 10 s. After 10 s, they were asked to spit the solution into the respective glass and wait 5 min before proceeding to the next solution/beverage. Next, in the first part, the temperature and pH of each expectorated solution/beverage were measured using a glass thermometer and a potentiometer (Hanna Instruments Inc., Woodstock, GA, USA), respectively. Then, for both tests, the samples were pipetted into 5 mL glass vials in the case of aqueous and hydroalcoholic solutions and into plastic 1.5 mL Eppendorf microtubes in the case of alcoholic beverages. All samples were coded and stored at −18 °C until enzymatic activity determination.

#### 2.3.3. Lipase and α-Amylase Activity Determination

Lipase enzyme activity was only measured for the first part of the study for the alcoholic beverages beer, wine, and brandy. The lipase enzyme assay kit (Abnova, Taipé, Taiwan) is based on a method in which -SH groups (thiol groups), formed from tributyrate-dimercaptopropanol (BALB) lipase cleavage, interact with 5,5 dithiobis (2-nitrobenzoic acid)—DTNB—to form a yellow-colored product. Color intensity was measured with the aid of a microplate reader (SPECTROstarNano, BMG LABTECH, Offenburg, Germany) at 412 nm. The measured absorbance is proportional to the enzyme activity in the sample [35]. The enzyme kit contained the reagents described in Supplementary Table S5. The detection threshold of the enzyme kit is in the range of 40 to 1600 U/L, with minimum and maximum values.

To prepare the working reagent, the colored reagent—DTNB—was mixed with assay buffer, and the flask was shaken to obtain a better homogenization. Then, 0.8 mL of BALB reagent was added. 

During the experimental procedure, 150 µL of water (H_2_O) was transferred to one well, and 150 µL of the calibrator (a substance not defined in the enzymatic kit—Table S1) was transferred to another well of the microplate. In the remaining wells, 10 µL of sample and 140 µL of working reagent prepared as mentioned above were added. The plates were covered, and the reaction mixture was homogenized. 

The OD_412nm_ was read in the microplate reader at 10 min (OD_10min_) and 20 min (OD_20min_) after the start of the reaction. The activity of the lipase enzyme was calculated according to Equation (1).

(1) $$\mathrm{Lipase}\;\mathrm{enzyme}\;\mathrm{activity}=\frac{\mathrm{OD}\;\left(20\min.\right)-\mathrm{OD}\;\left(10\min.\right)}{\mathrm{OD}\;\left(\mathrm{calibrator}\right)-\mathrm{OD}\;\left(H2O\right)}\times 735\;U/L$$

where OD_20min_/OD_10min_ represents the OD_412nm_ values of the sample at 20 min and 10 min, respectively, and OD_calibrator_ and OD_H2O_ represent the OD_412nm_ values of the calibrator and water at 20 min.

The number “735” is the equivalent activity (U/L) of the calibrator under the assay conditions.

For the determination of α-amylase, enzymatic kits from Biovision (Milpitas, CA, USA) were used. For each kit, a standard curve and respective equation of the straight line were drawn. 

The assay uses ethylidene–pNP–G7 as a substrate. Ethylidene–pNP–G7 is specifically cleaved by α-amylase. The final chromophore release is measured with the help of a microplate reader (SPECTROstarNano, BMG LABTECH, Offenburg, Germany) at a wavelength of 405 nm. The enzyme kit contained the reagents described in Table S5.

The α-amylase enzyme kit was previously stored at a temperature of −18 °C. 

For the use of the kit, the buffer was warmed to room temperature (±20 °C), and the samples and positive control were kept on ice during the assay. The nitrophenol standard curve (standard curve) was prepared with 0, 2, 4, 6, 8, and 10 µL of nitrophenol (2 nM) in duplicate, making a final volume of 50 µL with distilled water on the microplate. 

An absorbance/concentration curve was plotted for 0, 4, 12, 16, and 20 nmol/well of nitrophenol. In one of the microplate wells, 5 µL of positive amylase control and 45 µL of distilled water were added. 

Regarding the samples, 50 µL of each sample plus 50 µL of assay buffer and 50 µL of substrate mixture were added to each well, and two repetitions were performed. In the end, each microplate well contained a total volume of 150 µL. The content of the microplate was homogenized before reading.

Optical density (OD) was measured at a wavelength of 405 nm to obtain the OD T_0_. After eight minutes (T_1_), the OD was measured again. 

The standard nitrophenol curves were drawn from the results, and the equations were defined. The variation of OD [ΔOD = OD (T_1_) − OD (T_0_)] was calculated for the standard curve of nitrophenol, namely for the “y” of the first-degree equation, and the “x” of nitrophenol generated by the amylase between T_0_ and T_1_.

Finally, the α-amylase activity was calculated according to Equation (2).

(2) $$\mathrm{Amylase}\;\mathrm{enzyme}\;\mathrm{activity}\;\left(\frac{\mathrm{mU}}{\mathrm{mL}}\right)=\frac{B}{T\;\times \;V}\;$$

Where B is the nitrophenol amount from the standard curve (in nmol), T is the time between T_0_ and T_1_ (in min), and V is the pretreated sample volume added to the reaction well (in mL).

### 2.4. Beverage Sensory Evaluation through a Descriptive Analysis (DA) Sensory Test

Regarding the alcoholic beverages, in addition to performing the procedure described under Section 2.3.2, the tasters carried out a descriptive analysis [36]. For P1 (panel one), a tasting sheet for each class of beverage (beer, wine, and brandy) was constructed based on a list of attributes previously defined. The same process was carried out for P2 (panel two) for red wine and port wine, with new tasting sheets with descriptors specifically chosen for the work (Supplementary Table S6). In both tasting sessions, with P1 and P2, the tasters were asked to check the attributes they considered adequate to describe the different drinks.

### 2.5. Data Analysis

Data were presented as mean (M) and standard deviation (SD) when appropriate. The assumption of normality of distributions in each of the solutions/beverages studied was evaluated using the Shapiro-Wilk test. The assumption of homogeneity of variances was evaluated using the Levene test. To determine whether saliva influences the pH values of different alcoholic beverages, a one-sample t-test was performed. 

To assess whether there were significant differences in α-amylase and lipase activity between aqueous and hydroalcoholic solutions and to verify whether alcohol influenced the substances representing the basic tastes, a Kruskal–Wallis test and the Wilcoxon–Mann–Whitney test were used, respectively, since the conditions of normality and homogeneity of variances were not verified. To identify which basic tastes were significantly different, non-parametric pairwise comparisons were performed. 

To verify whether there were significant differences in alcoholic beverages (beer, wines, and brandies), in panel one (P1), in the enzymatic activity of lipase and α-amylase, a univariate analysis of variance (ANOVA) was executed, followed by a post hoc HSD Tukey test whenever possible.

To find out whether there were significant differences between red wine and port wine for panel two (P2) in the enzymatic activity of α-amylase, the Student’s t-test for independent samples was performed.

The statistical analyses were performed using SPSS Statistics (version 27.0), and in all analyses, a probability value of ≤0.05 was considered significant.

The sensory data obtained after the descriptive analysis (DA) test was graphically represented using the percentage of a citation for each descriptor. 

## 3. Results

### 3.1. Primary Taste Detection Thresholds in Aqueous and Hydroalcoholic Solutions

In the determination of the primary taste detection threshold in aqueous and hydroalcoholic solutions, there were some divergences in the answers given by the tasters. Nevertheless, in aqueous solutions, for 78% of the tasters, the sensation threshold of malic acid occurred at the concentration of 0.2 g/L and for about 22% at the concentration of 0.0 g/L. The threshold of perception occurred for 44.5% of the tasters at concentrations of 0.2 g/L or 0.5 g/L, while the remaining 11% achieved it at the concentration of 1.0 g/L. 

Regarding the threshold of sensation of citric acid and lactic acid, 90% of the individuals reported the existence of some substance at a concentration of 0.2 g/L and 10% at a concentration of 0.0 g/L. As for the perception threshold, for citric acid, 70% of the tasters detected the basic acid taste at the concentration of 0.2 g/L and 30% at the concentration of 0.5 g/L, while for lactic acid, the tasters were divided into the following percentages: 50% at the concentration of 0.2 g/L; 40% at a concentration of 0.5 g/L, and 10% at a concentration of 0.8 g/L. For lactic acid, two tasters mentioned that they detected a bitter basic taste and a salty basic taste instead of the acidic/sour taste.

For succinic acid, it was possible to verify that the sensation threshold of 90% of the tasters was at a concentration of 0.2 g/L, and the other 10% detected it at a concentration of 0.0 g/L. For the threshold of perception, the responses varied between a concentration of 0.2 g/L for 30% of the tasters and a concentration of 0.5 g/L for 70% of the individuals.

The salty taste was recognized by the panel, with a visible divergence in concentrations. Regarding the threshold of sensation of salty taste identification, 11% of the tasters reported a change in the taste of the solution at concentrations of 0.0 g/L and 0.5 g/L, while 33% reported a change at 1.0 g/L and the remaining 45% at 0.25 g/L. As for the threshold of perception, this was divided into different concentrations, with 11% of the taster’s detecting saltiness, represented by NaCl, at concentrations of 0.25 g/L and 4.0 g/L, 22% at 0.5 g/L and 2.5 g/L, and finally, 34% at of 1.0 g/L.

Concerning the basic sweet taste, 11% of the tasters noticed the existence of something in the solutions at 0.0 g/L, 7.5 g/L, and 10 g/L, and 33.5% in the solutions with 2.5 g/L and 5.0 g/L of sugar. As for the bitter taste, it was detected by 11% of the tasters at concentrations of 2.5 g/L, 7.5 g/L, and 13 g/L, and 33.5% of the tasters detected it at concentrations of 5.0 g/L and 10 g/L.

Concerning the primary taste detection threshold in hydroalcoholic solutions, for 75% of the tasters, the sensation threshold of succinic acid occurred at the concentration of 0.2 g/L and for about 25% at the concentration of 0.5 g/L. Regarding the threshold of perception, 17% of the tasters reported this at the concentration of 0.2 g/L, 33% at the concentration of 0.5 g/L, 42% at a concentration of 0.8 g/L, and the remaining 8% at a concentration of 1.0 g/L. 

For the threshold of sensation of lactic acid, 92% of the individuals reported the existence of some substance at the concentration of 0.2 g/L, while 8% perceived it at the concentration of 0.5 g/L. As for the perception threshold, for lactic acid, 25% of the tasters detected it at the concentrations of 0.2 g/L or 1.0 g/L, while for 42% of the tasters, it was detected at the concentration of 0.5 g/L, and 8% detected it at a concentration of 1.2 g/L.

As for the previous acids, for citric acid, the sensation threshold concentrations of 0.2 g/L and 0.5 g/L were perceived by 83% and 17% of the tasters, respectively. The perception threshold occurred for 42% of the tasters at 0.2 g/L, 33% at a concentration of 0.5 g/L, and 25% at a concentration of 0.8 g/L. 

For the determination of the threshold of the sensation of bitter taste, 92% of the tasters perceived the taste at a concentration of 0.5 g/L and 8% at a concentration of 2.5 g/L. The perception threshold, for 75% of the tasters, occurred at a concentration of 0.5 g/L, while for 17%, it occurred at 2.5 g/L, and for the remaining 8% at a concentration of 5.0 g/L. All tasters identified the basic bitter taste corresponding to tannins, and the sensation of astringency was also mentioned throughout the tasting.

For the sweet taste, 67% of the tasters had the sensation of sweetness at a concentration of 2.5 g/L, while 17% perceived it at a concentration of 5.0 g/L and 8% at concentrations of 7.5 g/L or 10 g/L. Regarding the sweet taste perception threshold, 33% of the panel detected it at concentrations of 2.5 g/L or 5.0 g/L, 17% at a concentration of 7.5 g/L, and 8% at concentrations of 10 g/L or 13 g/L.

Considering all these results, it was possible to choose average values of concentrations of compounds to prepare solutions for the next task: to determine the pH changes and the lipase and α-amylase activity after basic taste solutions, including the aqueous and hydroalcoholic solutions, came into contact with human saliva.

### 3.2. Determination of Temperature and pH Changes and Enzyme Evaluation in Aqueous/Hydroalcoholic Solutions and Beverages

#### 3.2.1. Temperature and pH

The temperature of aqueous/hydroalcoholic solutions and alcoholic beverages was measured before and after contact with the tasters’ oral cavity. It was found that the temperature of the solutions/drinks during the 10 s they were in the mouth increased by approximately 3.5 ± 1.0 °C.

To determine whether saliva influences the pH values of different alcoholic beverages, a one-sample t-test was performed after verifying the assumptions of data normality (*p* > 0.05). The pH values after contact with saliva are significantly higher than the initial values of the solutions (*p* < 0.05), except for the hydroalcoholic lactic acid solution (Table 2).

In both the aqueous solutions and hydroalcoholic solutions, the acidic solutions showed high resistance to pH changes. For example, in the citric acid solution, the pH changed from 3.19 to 3.39 and from 3.36 to 3.53 in aqueous and hydroalcoholic solutions, respectively. On the contrary, the aqueous solutions that stood out for having a lower resistance to changes in pH were the tannin and sodium chloride solutions, where an increase in pH values from 3.72 to 4.34 and from 4.96 to 5.92, respectively, was observed. In hydroalcoholic solutions, a significant change occurred in the tannin solution, in which the pH varied from 3.88 to 4.36, and in the sucrose solution, in which the pH varied from 6.19 to 6.70 (Table 2).

In alcoholic beverages, there were also significant differences in pH variation (Table 2). In colorless brandy and colored brandy, pH values were significantly higher after contact with human saliva, ranging from 4.30 to 4.84 (*p* < 0.001) in the case of colorless brandy and from 3.93 to 4.45 (*p* < 0.001) in the colored brandy. In contrast, for red and white wines, pH values were lower. In red wine, the pH dropped from 3.87 to 3.74 (*p* < 0.05); for white wine, the pH dropped from 3.37 to 3.22 (*p* < 0.05). However, the pH of the blonde beer did not significantly change (*p* = 0.941).

#### 3.2.2. Lipase and α-Amylase Enzymes Activity 

A Kruskal–Wallis test was performed to verify if there are differences in the enzymatic activity (lipase and α-amylase enzymes) in aqueous or hydroalcoholic solutions once the conditions of normality (*p* < 0.05) and homogeneity of variances (*p* < 0.05) were not verified.

The higher value of amylase activity was obtained in the sucrose and sodium chloride solutions (93.28 and 100.91 mU/mL, respectively), and the lower value was found in the tannin solution (0.49 mU/mL). The results obtained show significant differences in α-amylase activity in the presence of aqueous solutions (Table 3). According to the multiple pairwise comparisons of mean ranks, the differences per column are between tannin and sucrose solutions (*p* = 0.008), tannin and NaCl (*p* = 0.004), lactic acid and NaCl (*p* = 0.025), lactic acid and sucrose (*p* = 0.048), and citric acid and NaCl (*p* = 0.048).

In hydroalcoholic solutions, the results also indicated statistically significant differences in α-amylase activity. According to the multiple pairwise comparisons of mean ranks, the differences are, per column, between the solutions of tannin and lactic acid (*p* = 0.002), tannin and sucrose (*p* = 0.001), succinic acid and sucrose (*p* = 0.016), and tannin and citric acid (*p* = 0.014). The lowest value of enzymatic activity was found in the tannin solution (0.82 mU/mL), and the highest value was found in the sucrose solution (85.21 mU/mL).

To verify whether alcohol influenced α-amylase activity when tasters tasted solutions representing basic tastes, a Wilcoxon–Mann–Whitney test was performed (Table 4) once the conditions of normality (*p* < 0.05) and homogeneity of variances (*p* < 0.05) were not verified. It was possible to confirm that the differences observed between aqueous and hydroalcoholic solutions concerning lactic acid were significant (*p* = 0.025, Table 4). For this acid, aqueous solutions presented lower values of amylase activity than the hydroalcoholic solution.

To verify if there are differences in the enzymatic activity (lipase and α-amylase enzymes) in alcoholic beverages tasted by P1 (beer, wines, and brandies), an ANOVA was performed once the conditions of normality (*p* > 0.05) and homogeneity of variances (*p* > 0.05) were verified. The analysis of the results for alcoholic beverages for the α-amylase enzyme revealed that in at least one of the beverages, the mean value is significantly different from the others (F_(3, 5)_ = 56.59; *p* < 0.001) (Table 5). According to the post hoc test, the colorless brandy induced a significantly higher α-amylase enzyme activity (148.11 mU/mL) compared to the other beverages (*p* < 0.001). Similarly, red wine elicited greater enzyme activity (13.84 mU/mL) than white wine (4.15 mU/mL, *p* = 0.006) and blonde beer (2.03 mU/mL, *p* < 0.001) (Table 5).

Regarding the lipase enzyme activity, results from the aqueous and hydroalcoholic solutions were not considered for analysis because they were below the detection threshold of the lipase enzymatic kit (40 U/L). Only in the citric acid hydroalcoholic solution (Table 3) was it possible to observe a measurable lipase activity (57.95 U/L). However, results for lipase were obtained after saliva contact with alcoholic beverages, except for red wine. The higher enzymatic activity was observed for color brandy and white wine (400.80 and 270.65 U/L, respectively), and the lower enzymatic activity was observed for colorless brandy (41.49 U/L). Due to the considerable variations in the standard deviation, the differences were not significant (Table 5).

To verify if there are differences in the enzymatic activity (α-amylase) in alcoholic beverages (red wine and port wine, tasted by P2), a Student’s t-test for independent samples was performed. The results indicate that the differences found were significant (*p* = 0.005), as shown in Table 6. Furthermore, the amylase values of port wine are significantly higher than those found in red wine (*p* = 0.003).

### 3.3. Sensory Profile of the Alcoholic Drinks Determined by Descriptive Analysis (DA) Sensory Test

**Figure 1:**
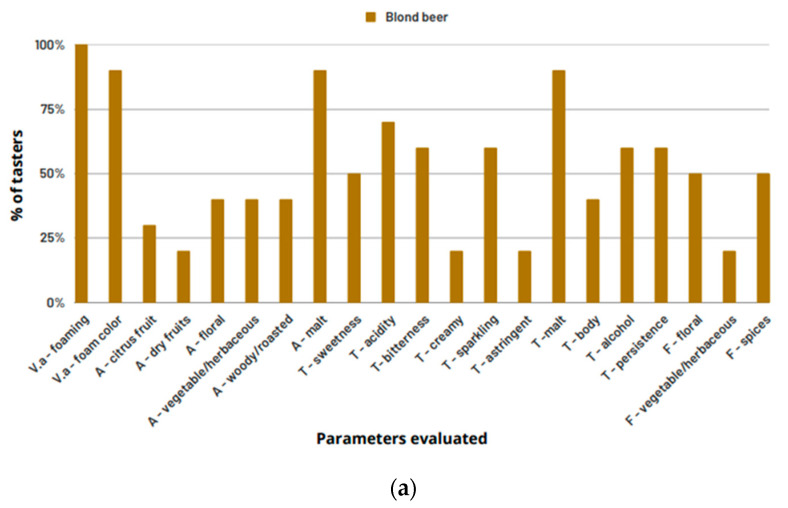

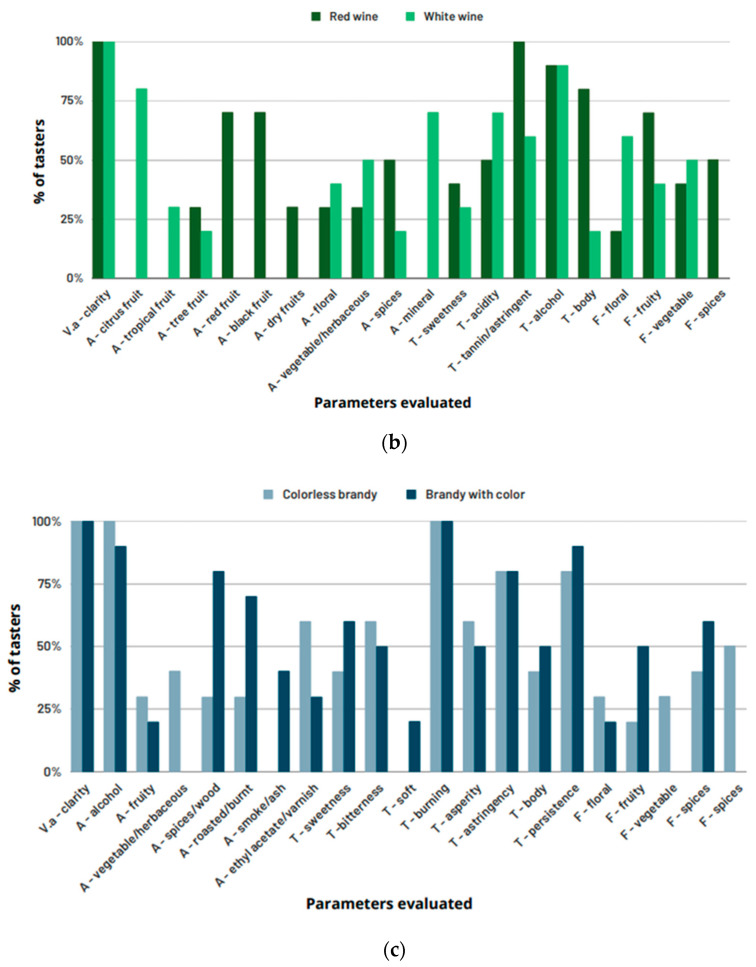
Percentage (%) of P1 tasters by parameters evaluated for beer (**a**), wines (**b**), and brandies (**c**) (Va—visual aspect; A—aroma; T—taste/texture; F—flavor).

Figure 1 shows the percentage of citations of each descriptor obtained after data analysis of the DA test tasting report for beer, wines, and brandies obtained with the help of the first panel (P1) composed of 11 individuals. Descriptors with percentages inferior to or equal to 10% were discarded. Despite the reduced number of tasters, the DA test was chosen because it was only intended to verify which attributes, from the list of attributes presented for each type of beverage, were identified by the tasters. For the blonde beer (Figure 1a), the descriptors that present the higher percentage of citations (higher than 60%) are “foaming” and “foaming color”, “malt aroma”, “acidity”, “bitterness”, “sparkling”, the mouthfeel sensation of bubbles, and “malt taste”. 

As can be seen in Figure 1b, the tasters perceived the red wine as more “fruity” (red fruit, black fruit, dried fruit sensations) and “sweeter” than the white wine. In red wine, the descriptors “tannin/astringent”, “body”, and “spices” also presented a percentage of citation higher than 60%, and the “spicy” sensation was only mentioned in the red wine. However, white wine was felt as having more “citrus” and “tropical fruits” and more “mineral”, “acidic”, and “floral” flavors.

Regarding the brandies (Figure 1c), although the alcoholic degree is similar for both drinks (41–40%, *v*/*v—*Table 5), the colorless brandy (Grappa) was perceived as having more “alcohol” and with a “vegetable/herbaceous” aroma. The varnish aroma of the descriptor “ethyl acetate” was also mentioned by twice as many tasters in the colorless brandy. The colorful brandy (Aguardente Velha) was characterized by the aromas “spices/wood”, “roasted/burnt”, “smoke/ash”, and was also perceived to be more “sweet”, “persistent”, “fruity, and “spiced”. 

**Figure 2:**
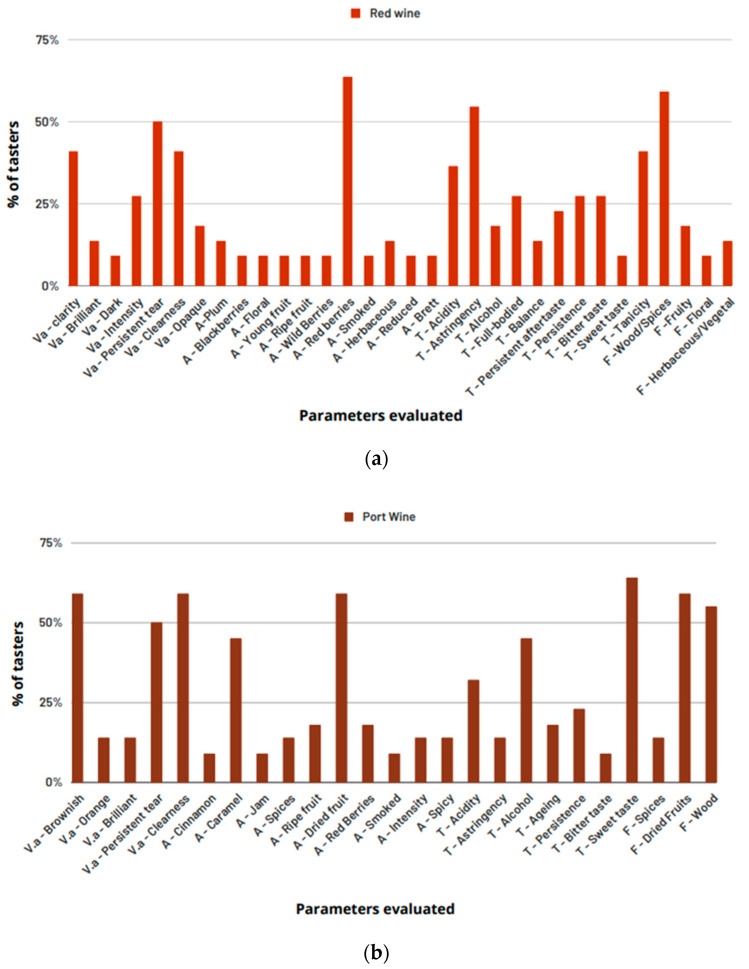
Percentage (%) of P2 tasters by parameters evaluated for red wine (**a**) and port wine (**b**) (Va—visual aspect; A—aroma; T—taste/texture; F—flavor).

To better understand what the tasters’ reaction was regarding the sensory analysis of alcoholic beverages, a more specific descriptive analysis was carried out for red wine (with more descriptors to be evaluated), as shown in Figure 2a, and another typical Portuguese beverage was taste-tested, port wine, as shown in Figure 2b. For this second sensory analysis, a second panel (P2) of 22 tasters was used.

Concerning red wine, it can be seen, once again, that the most quoted characteristics were “astringent”, “tannin”, and “wood/spices”. “Red berries” (>60%) and “acidity” (>35%) also stand out. Port wine is a fortified wine with very distinctive characteristics. The most mentioned attributes were “dried fruits”, “sweet taste”, and “wood”, with more than 55%, and “caramel” and “alcohol”, with approximately 65%.

## 4. Discussion

### 4.1. Sensation and Perception Threshold Determination

In some cases, the panelists felt the presence of a taste even in the absence of the substance in an aqueous solution. These results can be explained by considering several aspects: (i) it was possible that the concentration of the taste was too low for the assessor to correctly identify what taste it was; (ii) the latest meal that tasters ate (one to two hours before the tasting) may have influenced their taste perception. During the tasting session, the members of the panel described the last meal they made to find possible explanations for future results. It was possible to verify that tasters who felt the presence of a taste even in the absence of the substance ate foods with strong and persistent flavors, such as spicy sausages, hamburgers, and fries. The bibliography indicates that the taste of “meat” also evokes other mouth sensations such as “astringency” and “succulence” [37,38]. Regarding fried potatoes, more specifically French fries, they can bring quite complex flavors that come from frying [39]. A high lipolytic activity caused by the ingestion of these foods leads to an increase in the perception of fat and its aromatic compounds [11]. Studies also have shown that xenobiotic-metabolizing enzymes of saliva can be overexpressed after the consumption of certain foods rich in bioactive molecules [40], namely with salivary glutathione transferases, aldehyde dehydrogenase, and NADPH quinone oxidoreductases, which are overexpressed after the consumption of coffee or broccoli [41]. Thus, it is possible to infer that the food eaten two hours before the tasting can have a considerable impact on the results obtained, as all the taste sensations can last for more than an hour after the meal in the mouth. 

Another explanation for these strange results may be related to the analytical composition of the water—Caldas de Penacova—used in the preparation of the solutions. This water has a pH of 5.2 ± 0.4 and can be felt as slightly acidic, eliciting the sensation of “acid taste” even if no acid is present in the water.

Moreover, some confusion of perceptions was acknowledged. For instance, one taster, when detecting malic acid, classified it as having a “bitter taste”. The reason for the wrong classification may be related to the fact that the taster confuses the “sour taste” with the “bitter taste”, which reveals the importance of training the panelists and allowing them to familiarize themselves with the basic tastes. Furthermore, past a certain level of sourness, perception may be altered, and it has been known for many years that some people are extremely sensitive to the taste of bitter substances, while others perceive little or no bitter taste [42]. Another reason that can explain the confusion regarding the perception of the several tastes may be the fact that the acids studied, in addition to acidity, may confer characteristics such as bitterness and astringency to the solutions [43]. Furthermore, according to some authors, in addition to activating H^+^ ion receptors, acids also can stimulate nociceptive receptors that are connected to the nerve endings of the trigeminal nerve, which is responsible for sensations such as astringency [44]. 

In addition, tannin solutions were always accompanied by a feeling of astringency, a strong sensation characterized by roughness and dryness in the oral cavity. The feeling of astringency is produced by the binding and precipitation of salivary proteins and phenolic compounds, such as tannins [45]. Furthermore, we must not forget that the state of mind the tasters faced at the moment of the test, and the emotions they felt at that moment, could also be the cause of a change in sensation and perception [46]. 

In hydroalcoholic solutions, besides all the reasons mentioned above, ethanol becomes the most important factor for the difference in the responses obtained. Ethanol is an oral chemosensory stimulus and has complex sensorial attributes that are detected by multiple sensorial receptors and afferent fibers [2,47]. Neurophysiological studies have demonstrated a positive association between responses to alcohol and sweet stimuli in nerve fibers [48]. Several authors have described the sweet taste of ethanol in an aqueous solution containing low levels of ethanol (0–4% alc. vol.) [49,50], while others show that increasing the ethanol content of red and white wines to cover a range generally observed in dry wines (from 12 to 14% *v*/*v*) revealed no modification of the perception of wine sweetness, suggesting that ethanol has no direct effect on the sweet taste of wine [51]. In our results, the percentage of panelists that detected sweetness in aqueous and hydroalcoholic solutions was similar for the same sugar concentration, so, indeed, ethanol did not influence the results.

According to the literature, ethanol also has a strong effect on bitter taste sensitivity. A bitter taste and a burning sensation have been associated with higher levels of ethanol (10–22% *v*/*v*) [49,50], while more recent studies [51] showed that ethanol was not directly responsible for the perceived bitterness in white wine. In our work, the bitter taste elicited by the tannin solutions was perceived at lower concentrations in hydroalcoholic solutions than in aqueous solutions. Furthermore, as was mentioned, all tasters identified the bitter taste corresponding to tannin and perceived the solution astringency, contrary to what was mentioned by McRae et al. [52], who referred to the interference of ethanol with hydrophobic interactions between proteins and tannins, which may lead to a reduction of tannin precipitation and a decreased astringent sensation.

### 4.2. Influence of Saliva on the pH Variations

Saliva, like other body fluids, has a buffering capacity that allows it to absorb or release hydrogen ions (H^+^) to minimize changes in their concentration, that is, in the pH value [11,53]. The differences obtained in the pH values before and after contact with the human saliva allowed us to conclude that saliva influences the change in pH in alcoholic beverages in aqueous and hydroalcoholic solutions. An exception was seen for the solution with lactic acid in a hydroalcoholic medium. This acidic solution was the only one that did not show a significant difference in terms of pH change after the expectoration, that is, after contact with human saliva. Possible explanations for what happened may be related to lactic acid being a milder acid [54], being naturally present in the oral microflora [55] and having a pKa of about 3.85 (in the range of 19–23 °C), which can lead to a lower saliva–hydroalcoholic solution complex pH value [56].

When analyzing what occurred in the alcoholic beverages, we verified that there were divergences in the pH values that varied according to the beverage analyzed. In the case of brandies, the contact time of the drink with saliva and its buffering capacity was, once again, a limitation for the greater action of the pH of the saliva in these drinks. On the other hand, the slight decrease that occurred in the pH of red and white wine can be explained by considering a drink made up of different acids and in different concentrations. These acids, in particular, tartaric, malic, and citric acids, are responsible for limiting the pH of the wine as well as giving it a buffering capacity, which can be effective depending on the acidic components that are present [53], capable of causing an inhibition of the expected effect on the impact of saliva pH.

Regarding the blonde beer, the pH remained unchanged. Interestingly, in the blonde beer, the descriptor “foaming” presented a high frequency of citation, showing that beer has a high foam formation. According to Dysvik et al. [57], pH has a very complex effect on the foaming properties of beer, and higher pH values may be associated with higher foam production. Indeed, the pH of beer changes during the brewing process. First, we can consider that water will have a pH of over 7; when combined with crushed malt, the pH of the grain and water mixture drops considerably compared to the initial pH of the water alone. This observed pH decrease is the result of the precipitation of phosphates and amino acids derived from the malt. Phosphates, such as phosphoric acid, will disassociate. The presence of other minerals within the brewing water can interfere with the pH decrease during the brewing process. Specifically, the carbonate and bicarbonate ions associated with temporary water hardness can act as buffers to pH decrease. Usually, the pH of an infusion mash is around 5.2–5.6 [58]. During fermentation, the pH continues to drop. Yeast cells take in ammonium ions (which are strongly basic) and excrete organic acids (including lactic acid). Therefore, the biggest drop in pH is caused by fermentation and the acids, namely lactic acid, that is formed [58]. The beer presented to the tasters showed a high and persistent foam, following its higher pH (4.34), when compared with white and red wine’s pH (3.37 and 3.87, respectively). As the pH is closer to the pH of saliva, it is natural that the pH change becomes more difficult within 10 s of contact with saliva. On the other hand, lactic acid is one of the acids more present in beer [57] and is also one of the most important components responsible for the perceptible acidity of this drink (Figure 1a—descriptor “acidity”). Additionally, lactic acid is a much milder acid than tartaric and malic acid. Furthermore, the pKa of lactic acid can lower the pH of the saliva-beer complex to values very close to the beer pH.

### 4.3. Enzymatic Activity Elicited by Aqueous and Hydroalcoholic Solutions and Alcoholic Beverages

The salivary α-amylase is an endoglycohydrolase encoded by the gene *Amy1*. It hydrolyzes internal α-1,4-glucoside bonds of starch to the disaccharide maltose and moderate-length oligosaccharides called limit dextrins. These products adhere to chewed food and hold the bolus together for swallowing [59]. Human salivary amylase is inactivated in the acid pH of the gastric lumen [60] but more stable in the presence of the mouth saliva pH or in solutions where the pH is closer to the natural saliva pH, such as in the sucrose solutions tasted (pH between 5.67 and 6.19 in aqueous and hydroalcoholic solutions, respectively, Table 2). The high α-amylase activity in the sucrose solutions, both aqueous and hydroalcoholic (Table 3), reinforced that α-amylase is protected when in contact with these solutions and able to aid in the digestion of carbohydrates and starch [61]. Moreover, the acidic solutions (succinic, citric, lactic, and malic acids) in the two stock solutions (aqueous and hydroalcoholic) also elicited a relevant activity of the α-amylase enzyme (Table 3). The human body tends to increase saliva flow to dilute the acidity and thus protect itself [62], and the increase in saliva flow implicates an increase in the concentration of the α-amylase enzyme, although some acid inactivation may occur due to the lower pH of the acidic solutions, which explains why the activity of α-amylase is higher in sucrose solution than in acid solutions, despite the increase in saliva flow.

Regarding the low enzyme activity observed in tannin solutions (0.49 and 0.82 mU/mL in aqueous and hydroalcoholic solutions, respectively), the attraction between tannin and the α-amylase enzyme is reflected in the precipitation of the tannin—α-amylase complex, which may be responsible for the inactivation of the enzyme [63,64,65,66].

Regarding the salty taste, in aqueous solutions, the sodium chloride solution showed the greatest activity of the α-amylase enzyme (100.91 mU/mL, Table 2). These results are probably due to the properties of the salt, which, in addition to reducing the adsorption of proteins by the glass flasks where the saliva-solutions samples were stored, also has chloride ions, which are an essential cofactor for the enzyme’s activity [64].

Overall, aqueous solutions elicited lower enzyme activity compared to the hydroalcoholic solutions (Table 3 and Table 4). These differences may be due to the ethanol present in the hydroalcoholic solutions. This compound causes stress to the taster due to the heat and burning felt when tasting, triggering an increase in the production of α-amylase [16]. In lactic acid, this variance is considerably high as, in the hydroalcoholic solution, a many-fold increase in α-amylase activity occurred when compared with the activity observed in the aqueous solution.

Thus far, ‘food–saliva interactions’ has been used as a general term to refer to all possible interactions between food ingredients and saliva compounds. An example is the work of Zhao et al. [67], which studied the interaction between human salivary α-amylase and sorghum procyanidin tetramer. Recently, food–saliva interactions have also been investigated in beer [68], wine [69], and other food systems [70].

Regarding beer, Ramsey et al. [68] hypothesized that ethanol and saliva interactions, namely, ethanol and α-amylase interactions, directly impact the sensory and flavor properties of beer. These authors found that ethanol has a subtle inhibitory effect on the binding of hydrophobic compounds to α-amylase, thereby increasing their headspace concentration in the 5% (*v*/*v*) alcoholic beers as compared to the 0% beers. The sensory data did not show significant differences in orthonasal perception between these two kinds of beer, yet retronasal results showed that 0% lager was perceived as maltier with reduced fruitiness, sweetness, fullness/body, and alcohol-warming sensation (*p*  <  0.05). In our work, the blonde beer with 4.9% (*v*/*v*) alcohol content was characterized by the sensory descriptors of foaming, malt aroma, acidity, bitterness, and sparkling, the mouthfeel sensation of bubbles, and malt taste. This beer induced lower α-amylase enzyme activity when compared to red wine, probably due to the lower alcoholic concentration content when compared with the other beverages. 

Red wine is perceived to be more fruity and sweeter, and brandy aged in oak wood is considered sweet, persistent, fruity, and spiced; these drinks induced a greater activity of the α-amylase enzyme. This phenomenon probably occurred because the vinification process of red wine—which allows for an increase in reducing sugars—and of colored spirits—which makes it less aggressive and with more roasted and caramelized aromas—caused a positive synergistic effect between these drinks and the activity of the enzyme.

Small molecules, such as ethanol, influence the compound retention effect and the hydrolysis function by reacting with salivary amylase [71]. Interestingly, despite its alcoholic concentration (41% (*v*/*v*)), the colorless brandy (bottle-aged) showed significantly higher α-amylase activity compared to the other analyzed beverages (Table 5), showing that something must have triggered a higher production of amylase once some of it reacted with ethanol. According to several authors [64,65,66], α-amylase activity increases in response to stress, whether physical or psychological. Thus, the high alcohol content (41.0 %, *v*/*v*) of the brandy and the fact that it caused an alcohol sensation in the mouth and sensations of varnish and vegetable/herbaceous aroma (DA test, Figure 1c) when it was tasted may indicate an increase in stress and anxiety on the part of the taster as a physiological response to tasting. The roasted and caramelized aromas and flavors presented in the colored brandy came from the contact it had with the wood during the manufacturing process. These changes produced by the wood make the burning sensation less intense, and consequently, the tasters preferred this brandy compared to the colorless one, meaning that it probably did not cause so much stress when tasting, leading to lower α-amylase activity when compared to the colorless brandy (Table 5).

To confirm the hypotheses and obtain more reliable data, a second panel (P2) with a larger number of tasters was used, and new tests and saliva samples were taken to assess amylase enzyme activity. The beverages in question were again a red wine and, this time, a port wine. It was possible to verify that the enzyme activity was higher in port wine (with 19% (*v*/*v*) alcohol content) compared to red wine (13.5% (*v*/*v*)), as seen in Table 6.

The port wine used was a tawny wine. This style of wine presents a sugar content of 40g—65 g/L, so it is a very sweet wine. Its aroma ranges from jam to dried fruits, such as hazelnuts and walnuts [72]. The aging in wood gives the fortified wines some touches of caramel and wood resulting from the Maillard reactions [72,73]. All these descriptors were also mentioned by the tasters. These characteristics, together with the alcohol and acidity also detected by the tasters, as seen in Figure 2a, tend to reinforce the idea of increased α-amylase activity in this type of beverage.

The spectrophotometric methods for the determination of lipase activity make use of synthetic lipase substrates transformed upon enzyme-catalyzed hydrolysis into products able to be detected spectrophotometrically. However, this method was not efficient in determining the lipase activity after tasting aqueous and hydroalcoholic solutions, and the results were not considered for analysis because they were below the threshold for detection of the lipase enzyme kit (40 U/L). Moreover, DTNB (5,5 dithiobis (2-nitrobenzoic acid) can also react with free thiol from salivary proteins, leading to background variation from one saliva sample to another depending on the individual salivary protein concentration [74]. Only in the citric acid hydroalcoholic solution was it possible to observe a measurable activity, that is, a value within the detection threshold of the lipase enzyme kit. This situation may be related to the fact that the lipase enzyme is present in low concentrations in human saliva [62], and, therefore, the lipase enzyme activity kit used in this study was not the most suitable for measuring its activity.

Regarding alcoholic beverages, positive results regarding the activity of this enzyme were obtained after saliva contact with these beverages, except for red wine. One possible explanation for the lipase activity being measurable in alcoholic beverages is the fact that the beverages are complex and fatty acids are part of their composition. These fatty acids come from raw material, the fermentative activity of yeasts, and from the yeast themselves once they release fatty acids into the medium (wine or beer) after finishing the fermentation process. Nonetheless, and as referred to by Stoytcheva et al. [75], a common disadvantage of the spectrophotometric methods is the low substrate specificity of the enzyme towards the synthetic substrate analogs, which can cause some discrepancies in the results.

## 5. Conclusions

The composition and buffering capacity of saliva influence the perception of flavor. The pH of aqueous hydroalcoholic solutions and alcoholic beverages remained close to their initial values and not to the pH values of human saliva, that is, between 6.2 and 7.4. Thus, it is possible to conclude that the buffering capacity of saliva is not sufficient to maintain a constant pH after contact with the solutions/beverages.

The α-amylase activity significantly increased when solutions contained acids and/or ethanol and decreased in the presence of tannin, probably due to its precipitation caused by tannin–protein interactions.

When comparing red wine with port wine, we also verified a higher α-amylase activity in port wine, probably due to its sweet taste and higher alcoholic degree. 

Regarding the activity of the lipase enzyme, it was observed that the existing concentration in human saliva may be below the detection thresholds of the enzyme kit used. However, in the case of alcoholic beverages, lipase activity was visible.

More sensitive kits, or other analytical methods, are needed to improve saliva enzyme activity determinations.

Looking ahead, a wider study not only of other enzymes/proteins that are relevant to the interaction of human saliva with beverages but also with various types of wine and alcoholic and non-alcoholic beverages will allow a better understanding of saliva and beverage interactions.

## Figures and Tables

**Table 1:** Concentrations used for the preparation of aqueous and hydroalcoholic solutions (ethanol 12%, *v*/*v*) for enzyme activity determinations.

Taste Descriptor	Representative Substance	Concentration (Aqueous Solutions), g/L	Concentration (Hydroalcoholic Solutions), g/L
Sweet	Sucrose	10.0	5.0
Salty	Sodium chloride	1.0	N.p.
Acid	Malic acid	0.2	N.p.
	Citric acid	0.2	0.2
	Lactic acid	0.2	0.5
	Succinic acid	0.5	0.8
Bitter/astringency	Tannin	0.5	0.5

N.p.—not performed.

**Table 2:** pH of aqueous solutions, hydroalcoholic solutions, and alcoholic beverages before contact with tasters’ saliva. Descriptive measures (M ± SD) and univariate effects of aqueous, hydroalcoholic, and alcoholic beverages after contact with saliva. In the case of alcoholic beverages, the alcohol content (%, *v*/*v*) corresponding to each one is shown.

	Solutions/Beverages Before Contact with Saliva			The pH of the Solutions/Beverages after Contact with Saliva		
Aqueous solutions	**Taste descriptor**	**Representative Substances**	**pH**	**pH** **(M ± SD)**	***t***	***p***
	Sour	Malic acid ^1^	3.25	3.37 ± 0.042	7.378	<0.001
		Succinic acid ^1^	3.30	3.54 ± 0.047	13.221	<0.001
		Citric acid ^1^	3.19	3.39 ± 0.059	8.862	<0.001
		Lactic acid ^1^	3.31	3.49 ± 0.052	8.984	<0.001
	Bitter	Tannin ^1^	3.72	4.34 ± 0.349	4.732	0.003
	Sweet	Sucrose ^1^	5.67	5.98 ± 0.175	4.727	0.003
	Salty	Sodium chloride ^1^	4.96	5.92 ± 0.195	13.008	<0.001
Hydroalcoholic Solutions	Sour	Succinic acid ^1^	3.36	3.51 ± 0.124	3.403	0.011
		Citric acid ^1^	3.36	3.53 ± 0.081	5.901	0.001
		Lactic acid ^1^	3.26	3.29 ± 0.047	1.504	0.176
	Bitter	Tannin ^1^	3.88	4.36 ± 0.107	12.716	<0.001
	Sweet	Sucrose ^1^	6.19	6.70 ± 0.189	11.630	<0.001
Alcoholic beverages	**Type of Beverage**	**Alcohol (%, *v*/*v*)**	**pH**	**pH** **(M ± SD)**	***t***	***p***
	Colorless brandy or Grappa (Bottle-aged)	41.0 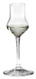	4.30	4.84 ± 0.249	6.824	<0.001
	Color brandy or Aguardente Velha (Wood-aged)	40.0 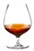	3.93	4.45 ± 0.268	6.091	<0.001
	White wine	13.0	3.37	3.22 ± 0.123	−3.902	0.004
	Red wine	13.5 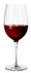	3.87	3.74 ± 0.119	−3.400	0.008
	Blonde beer	4.9 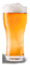	4.34	4.34 ± 0.066	0.076	0.941

^1^ Concentrations referred to in Table 1; *t*—observed value of test statistics; *p*—probability value.

**Table 3:** Descriptive measurements and results of the Kruskal–Wallis test of enzymatic activity (lipase and α-amylase enzymes) in aqueous and hydroalcoholic solutions.

		Succinic Acid M ± SD	Citric Acid M ± SD	Lactic Acid M ± SD	Malic Acid M ± SD	Sucrose M ± SD	Tannin M ± SD	Sodium Chloride M ± SD	$\chi_{kw}^{2}$	*p*
**Aqueous**	α-Amyl. mU/mL	49.6 ± 36.8 b	24.05 ± 31.5 b	9.33 ± 15.1 b	34.96 ± 56.4 b	93.28 ± 22.5 c	0.49 ± 0.86 a	100.91 ± 20.8 c	13.11	0.041
	Lipase U/L	-*	-*	-*	-*	-*	-*	-*	-*	-*
**Hydroalcoholic**	α-Amyl. mU/mL	44.37 ± 18.7 a	63.89 ± 21.1 b	75.79 ± 17.5 b	N.p.	85.21 ± 13.4 b	0.82 ± 1.3 a	N.p.	17.89	0.001
	Lipase U/L	-*	57.96 ± 46.0	-*	N.p.	-*	-*	N.p.	-*	-*

* Results from the aqueous and hydroalcoholic solutions for the lipase enzyme were not considered for analysis because they are below the threshold for detection of the lipase enzyme kit (40 to 1600 U/L); N.p.—not performed. *p—*probability value. For each type of solution (aqueous or hydroalcoholic) and the same enzyme, values with the same letter, per column, are not significantly different (non-parametric pairwise comparisons).

**Table 4:** Comparison of compounds in aqueous and hydroalcoholic solutions and results of the Wilcoxon–Mann–Whitney test of the enzymatic activity of α-amylase (mU/mL).

Compounds	Aqueous M ± SD	Hydroalcoholic M ± SD	Z	*p*
Succinic acid	49.67 ± 36.79	44.37 ± 18.68	−0.149	0.881
Citric acid	24.05 ± 31.46	63.89 ± 21.09	−1.640	0.101
Lactic acid	9.33 ± 15.12	75.79 ± 17.53	−2.236	0.025
Sucrose	93.28 ± 22.47	85.21 ± 13.35	−0.447	0.655
Tannin	0.49 ± 0.86	0.82 ± 1.29	−0.764	0.445

Z—observed value of test statistics; *p—*probability value.

**Table 5:** Descriptive measurements and univariate effects of enzymatic activity (lipase and α-amylase enzymes) in alcoholic beverages (brandies, wines, and beer).

	Colorless Brandy M ± SD	Color Brandy M ± SD	White Wine M ± SD	Red Wine M ± SD	Blonde Beer M ± SD	F	*p*
α—Amylase mU/mL	148.11 ± 31.15 c	44.18 ± 38.68 ab	4.15 ± 4.78 a	13.84 ± 4.85 b	2.03 ± 1.61 a	56.59	<0.001
Lipase U/L	41.49 ± 1.58	400.80 ± 135.88	270.65 ± 220.89	-*	63.84 ± 19.29	4.027	0.205

* Result not considered for analysis because it was below the threshold for detection of the lipase enzyme kit (40 to 1600 U/L). F–observed value of test statistics; *p—*probability value. Values with the same letter are not significantly different (post hoc Tukey’s HSD test).

**Table 6:** Descriptive measurements and univariate effects of enzymatic activity (α-amylase) in alcoholic beverages (red wine and port wine) and Student’s t-test for independent samples.

	Red Wine M ± SD	Port Wine M ± SD	t	*p*
α-Amylase (mU/mL)	28.62 ± 49.54	72.55 ± 65.64	−2.90	0.005

*t—*observed value of the test statistics; *p—*probability value.

## Data Availability

Data is contained within the article or supplementary material.

## Supplementary Materials

The following supporting information can be downloaded at: https://www.mdpi.com/article/10.3390/foods12051018/s1, Table S1: Concentrations and pH [mean (M) ± standard deviation (SD)] of solutions used to determine sensory thresholds of taste attributes sweet, salty, bitter, and astringency sensation, Table S2: Concentrations and pH [mean (M) ± standard deviation (SD)] of solutions used to determine sensory thresholds of acid taste attribute, Table S3: Concentrations and pH [mean (M) ± standard deviation (SD)] of 12% (*v*/*v*) hydroalcoholic solutions used to determine sensory thresholds of taste attribute sweet, bitter, and astringency sensation, Table S4: Concentrations and pH [mean (M) ± standard deviation (SD)] of 12% (*v*/*v*) hydroalcoholic solutions used to determine sensory thresholds of basic acid taste attributes, Table S5: Composition of the lipase and α-amylase enzyme kits, Table S6: Descriptors analyzed in the Descriptive Analysis (DA) sensory test of Panel one (P1) for each of the beverages beer, wines and brandies. Descriptive Analysis (DA) sensory test of Panel two (P2) for red wine and Port Wine. V.a - Visual appearance; A- Aroma; T-Taste/Texture; F - Flavor (retronasal).
