# Supplementary material for: Exploring the Impact of α-Amylase Enzyme Activity and pH on Flavor Perception of Alcoholic Drinks
Source: Foods. 2023 Feb 27;12(5):1018. doi: 10.3390/foods12051018 (PMC10000705; doi:10.3390/foods12051018)
Supplement: Supplementary file 1 [file foods-12-01018-s001.zip › Table 1S.pdf]

Table S1. Concentrations and pH [mean (M) ± standard deviation (SD)] of solutions used to determine sensory thresholds of taste attribute sweet, salty, bitter, and astringency sensation.

Taste attribute	Representative substance	Concentration	pH (M ± SD)
Sweet g/L	Sucrose $C_{12}H_{22}O_{11}$	0.0	5.47 ± 0.021
		2.5	5.74 ± 0.005
		5.0	5.68 ± 0.003
		7.5	5.76 ± 0.004
		10.0	5.74 ± 0.004
		13.0	5.72 ± 0.000
		20.0	5.78 ± 0.002
Salty g/L	Sodium Chloride NaCl	0.0	5.47 ± 0.021
		0.25	3.81 ± 0.002
		0.5	4.82 ± 0.002
		1.0	5.60 ± 0.006
		2.5	5.60 ± 0.005
		4.0	5.24 ± 0.003
		6.0	4.62 ± 0.003
Bitter mg/L	Quinine Sulfate $C_{20}H_{24}N_2O_2$	0.0	5.47 ± 0.021
		0.5	5.93 ± 0.001
		1.0	5.99 ± 0.006
		2.5	5.46 ± 0.001
		5.0	5.19 ± 0.001
		7.5	5.25 ± 0.001
		10.0	5.28 ± 0.002
Bitter | Astringency g/L	Tannin	0.0	5.47 ± 0.021
		0.5	3.75 ± 0.004
		1.0	3.49 ± 0.005
		2.5	3.27 ± 0.006
		4.0	3.14 ± 0.013
		5.0	3.09 ± 0.004
		6.0	3.03 ± 0.004
