# Supplementary material for: Exploring the Impact of α-Amylase Enzyme Activity and pH on Flavor Perception of Alcoholic Drinks
Source: Foods. 2023 Feb 27;12(5):1018. doi: 10.3390/foods12051018 (PMC10000705; doi:10.3390/foods12051018)
Supplement: Supplementary file 1 [file foods-12-01018-s001.zip › Table 2S.pdf]

Table S2. Concentrations and pH [mean (M) ± standard deviation (SD)] of solutions used to determine sensory thresholds of acid taste attribute.

**Acids Concentrations**	**pH (M ± SD)**			
**(g/L)**	**Malic acid** $C_4H_6O_5$	**Citric** $C_6H_8O_7$	**Lactic** $C_3H_6O_3$	**Succinic** $C_4H_6O_4$
0.0	5.47 ± 0.021	5.47 ± 0.021	5.47 ± 0.021	5.47 ± 0.021
0.2	3.25 ± 0.004	3.16 ± 0.006	3.29 ± 0.002	3.51 ± 0.001
0.5	2.98 ± 0.000	2.93 ± 0.004	3.18 ± 0.005	3.31 ± 0.006
0.8	2.83 ± 0.003	2.42 ± 0.001	2.81 ± 0.004	3.17 ± 0.004
1.0	2.78 ± 0.007	2.88 ± 0.003	2.80 ± 0.000	3.10 ± 0.004
1.2	2.79 ± 0.014	2.75 ± 0.008	2.74 ± 0.001	3.05 ± 0.001
1.5	2.69 ± 0.003	2.68 ± 0.011	2.74 ± 0.001	3.01 ± 0.002
