# Supplementary material for: Exploring the Impact of α-Amylase Enzyme Activity and pH on Flavor Perception of Alcoholic Drinks
Source: Foods. 2023 Feb 27;12(5):1018. doi: 10.3390/foods12051018 (PMC10000705; doi:10.3390/foods12051018)
Supplement: Supplementary file 1 [file foods-12-01018-s001.zip › Table 3S.pdf]

Table S3. Concentrations and pH [mean (M) ± standard deviation (SD)] of 12% (v/v) hydroalcoholic solutions used to determine sensory thresholds of taste attribute sweet, bitter, and astringency sensation.

**Taste attribute**	**Representative Substance**	**Concentration (g/L)**	**pH (M ± SD)**
Sweet	Sucrose $C_{12}H_{22}O_{11}$	0.0	5.59 ± 0.035
		2.5	5.96 ± 0.001
		5.0	5.99 ± 0.005
		7.5	5.96 ± 0.001
		10.0	5.44 ± 0.005
		13.0	5.36 ± 0.004
		20.0	5.87 ± 0.003
Bitter׀Astringency g/L	Tannin	0.0	5.59 ± 0.035
		0.5	3.52 ± 0.001
		1.0	3.51 ± 0.004
		2.5	3.36 ± 0.004
		4.0	3.25 ± 0.004
		5.0	3.19 ± 0.004
		6.0	3.14 ± 0.004
