# Supplementary material for: Exploring the Impact of α-Amylase Enzyme Activity and pH on Flavor Perception of Alcoholic Drinks
Source: Foods. 2023 Feb 27;12(5):1018. doi: 10.3390/foods12051018 (PMC10000705; doi:10.3390/foods12051018)
Supplement: Supplementary file 1 [file foods-12-01018-s001.zip › Table 4S.pdf]

Table S4. Concentrations and pH [mean (M) ± standard deviation (SD)] of 12% (v/v) hydroalcoholic solutions used to determine sensory thresholds of basic acid taste attributes.

**Acids Concentrations**	**pH (M± SD)**		
(g/L)	**Citric** $C_6H_8O_7$	**Lactic** $C_3H_6O_3$	**Succinic** $C_4H_6O_4$
0.0	5.59 ± 0.035	5.59 ± 0.035	5.59 ± 0.035
0.2	3.26 ± 0.003	3.36 ± 0.006	3.59 ± 0.004
0.5	3.13 ± 0.003	3.01 ± 0.005	3.34 ± 0.003
0.8	2.83 ± 0.005	2.90 ± 0.004	3.22 ± 0.003
1.0	2.78 ± 0.004	2.84 ± 0.003	3.16 ± 0.002
1.2	2.71 ± 0.004	2.79 ± 0,003	3.12 ± 0.002
1.5	2.67 ± 0.001	2.77 ± 0.001	3.07 ± 0.000
