# Supplementary material for: Exploring the Impact of α-Amylase Enzyme Activity and pH on Flavor Perception of Alcoholic Drinks
Source: Foods. 2023 Feb 27;12(5):1018. doi: 10.3390/foods12051018 (PMC10000705; doi:10.3390/foods12051018)
Supplement: Supplementary file 1 [file foods-12-01018-s001.zip › Table 5S.pdf]

Table S5. Composition of the lipase and α-amylase enzyme kits.

Type of Kit	Components	Amount
**Lipase**<br>(Abnova, Taipé, Taiwan)	Assay Buffer – pH 8.5	15 mL
	Color reagent – DTNB<br>[5,5-dithio-bis-(2-nitrobenzoic acid)]	530 mg
	BALB Reagent<br>(dimercaptopropanol tributyrate)	1.0 mL
	Calibrator	2.0 mL<br>(equivalent to 735 U/L)
**α-Amylase**<br>(Biovision, Milpitas, US)	Assay Buffer	55 mL
	Substrate Mixing	5 mL
	Amylase positive control<br>(lyophilized)	1 vial
	Nitrophenol Standard (2nM)	150 µL
