# Supplementary material for: Exploring the Impact of α-Amylase Enzyme Activity and pH on Flavor Perception of Alcoholic Drinks
Source: Foods. 2023 Feb 27;12(5):1018. doi: 10.3390/foods12051018 (PMC10000705; doi:10.3390/foods12051018)
Supplement: Supplementary file 1 [file foods-12-01018-s001.zip › Table 6S.pdf]

Table S6. Descriptors analyzed in the Descriptive Analysis (DA) sensory test of Panel one (P1) for each of the beverages beer, wines and brandies. Descriptive Analysis (DA) sensory test of Panel two (P2) for red wine and Port Wine. V.a - Visual appearance; A- Aroma; T-Taste/Texture; F - Flavor (retro nasal).

Panel one (P1)			Panel two (P2)	
Beer	Wines	Brandies	Red wine	Port Wine
V.a - foaming<br>V.a - foam color	V.a - clarity	V.a - clarity	Va - clarity<br>Va - brilliant<br>Va - dark<br>Va - intensity<br>Va - persistent tear<br>Va - clearness<br>Va – opaque	V.a - brownish<br>V.a - orange<br>V.a - brilliant<br>V.a - persistent tear<br>V.a - clearness
A - citrus fruit<br>A - dry fruits<br>A - floral<br>A - vegetable /herbaceous<br>A - woody/roasted<br>A - malt	A - citrus fruit<br>A - tree fruit<br>A - tree fruit<br>A - red fruit<br>A - black fruit<br>A - dry fruits<br>A - floral<br>A - vegetable /herbaceous<br>A - spices<br>A - mineral	A -alcohol<br>A - fruity<br>A - vegetable/ herbaceous<br>A - spices/wood<br>A - roasted/burnt<br>A - smoke/ash<br>A - ethyl acetate/ varnish	A - plum<br>A - blackberries<br>A - floral<br>A - young fruit<br>A - ripe fruit<br>A - blackberry<br>A - wild Berries<br>A - red berries<br>A - smoked<br>A - herbaceous<br>A - reduced<br>A - brett	A - cinnamon<br>A - caramel<br>A - jam<br>A - spices<br>A - ripe fruit<br>A - dried fruit<br>A - red Berries<br>A - smoked<br>A - intensity<br>A - spicy
T - sweetness<br>T - acidity<br>T- bitterness<br>T - salty<br>T - creamy<br>T - sparkling<br>T - astringent<br>T - spicy<br>T -malt<br>T - body<br>T - alcohol<br>T – persistence	T – sweetness<br>T – acidity<br>T - tannin/ astringent<br>T – alcohol<br>T - body	T – sweetness<br>T -bitterness<br>T – soft<br>T – burning<br>T – asperity<br>T – astringency<br>T – body<br>T - persistence	T - acidity<br>T - astringency<br>T - alcohol<br>T - full-bodied<br>T - balance<br>T - persistent aftertaste<br>T - persistence<br>T - bitter taste<br>T - sweet taste<br>T - tanicity	T - acidity<br>T - astringency<br>T - alcohol<br>T - ageing<br>T - persistence<br>T - bitter taste<br>T - sweet taste
F – floral<br>F – fruity<br>F - vegetable/ herbaceous<br>F - spices	F – floral<br>F – fruity<br>F – vegetable/ herbaceous<br>F - spices	F – floral<br>F – fruity<br>F – vegetable/ herbaceous<br>F - spices	F -wood/spices<br>F -fruity<br>F - floral<br>F - vegetable/ herbaceous	F - spices<br>F – dried fruits<br>F - wood
